# Effect of temperature on the *Bulinus globosus — Schistosoma haematobium* system

**DOI:** 10.1186/s40249-017-0260-z

**Published:** 2017-05-01

**Authors:** Chester Kalinda, Moses J. Chimbari, Samson Mukaratirwa

**Affiliations:** 1 School of Nursing and Public Health, College of Health Sciences, Howard College Campus, University of KwaZulu-Natal, Durban, South Africa; 2 School of Life Sciences, College of Agriculture, Engineering and Science, Westville Campus, University of KwaZulu-Natal, Durban, South Africa

**Keywords:** *Bulinus globosus*, Cercariae, Development rate, Fecundity, Schistosomiasis, *Schistosoma haematobium*, Temperature

## Abstract

**Background:**

Given that increase in temperature may alter host-parasite relationships, the anticipated rise in temperature due to global warming might change transmission patterns of certain diseases. However, the extent to which this will happen is not well understood.

**Methods:**

Using a host-parasite system involving *Bulinus globosus* and *Schistosoma haematobium*, we assessed the effect of temperature on snail fecundity, growth, survival and parasite development under laboratory conditions.

**Results:**

Our results show that temperature may have a non-linear effect on snail fecundity and snail growth. Snails maintained at 15.5 °C and 36.0 °C did not produce egg masses while those maintained at 25.8 °C laid 344 and 105 more egg masses than snails at 31.0 °C and 21.2 °C, respectively. Attainment of patency led to a reduction in egg mass production among the snails. However, the reduction in fecundity for snails maintained at 21.2 °C occurred before snails started shedding cercariae. Parasite development was accelerated at high temperatures with snails maintained at 31.0 °C reaching patency after three weeks. Furthermore, snail growth rate was highest at 25.8 °C while it was inhibited at 15.5 °C and reduced at 31.0 °C. Increase in temperature increased snail mortality rates. Snails maintained at 36.0 °C had the shortest survival time while those maintained at 15.5 °C had the longest survival time.

**Conclusions:**

We concluded that temperature influences fecunxdity, growth, survival and parasite development in the snail and thus dictates the time it takes the parasite to complete the life cycle. This has implications on transmission of schistosomiasis in the context of global warming.

**Electronic supplementary material:**

The online version of this article (doi:10.1186/s40249-017-0260-z) contains supplementary material, which is available to authorized users.

## Multilingual abstracts

Please see Additional file 1 for translations of the abstract into the five official working languages of the United Nations.

## Background

Schistosomiasis is a parasitic disease caused by blood fluke trematodes of the genus *Schistosoma* [1]. *Schistosoma haematobium* and *Schistosoma mansoni* are the two major species affecting people in southern Africa [1, 2]. Different stages of the disease cycle are affected by temperature [3]. Temperature influences the physiology, ecology, susceptibility of snails to infection and parasite pathogenicity [4–7]. Earlier studies [8–10] observed that *B. globosus*, the intermediate host snail of *S. haematobium*, had high thermal tolerance and adapted in areas with fluctuating temperature [11, 12]. This snail has also been observed to have marked increase in survival time at temperatures as high as 34.0 °C [6] while its optimal temperature for fecundity and growth has been shown to be 25.0 °C [11]. Furthermore, this temperature coincides with the optimal temperature range (22 – 27 °C) for disease transmission [13]. This suggests that the anticipated rise in temperature due to climate change may have serious implications on snail population size and schistosomiasis spread patterns.

The effect of temperature on the growth and reproduction of snails may influence the development rates of parasites and hosts [14]. Growing evidence suggests that under future global warming scenarios, host population dynamics may play vital roles in determining disease spread patterns and parasite output load [15]. Temperature promotes the development of hosts and parasites while reducing their survival [16] suggesting that its net effect on host development and population size may be important in determining disease spread and parasitism levels. Contrasting observations on the potential effects of temperature rise on snails have been made. Pedersen et al. [17] and Martens et al. [18] suggested a possible reduction in habitat suitability and disease transmission while Zhou et al. [19] and McCreesh et al. [20] suggested that a rise in temperature may lead to an increase in suitable habitats and schistosomiasis prevalence. Furthermore, Nelson et al. [21] suggested that adaption of snails to higher temperatures may affect their susceptibility to infection.

In view of these conflicting potential outcomes of climate change on schistosomiasis risks, understanding the possible effects of temperature on snails through mechanistic approaches may show the broad potential impacts of climate change on the transmission of schistosomiasis. This will also assist in understanding the potential influence of temperature changes on patterns of snail growth, fecundity, survival and parasite development. Using *B. globosus*, we experimentally assessed the effect of *S. haematobium* infection and temperature on the fecundity, survival and growth of the intermediate host snails and production of cercariae*.* The study developed a mechanistic host-parasite model for understanding the potential impacts of future temperature rise on the snail-trematode system.

## Methods

### Breeding of experimental animals

Sexually mature *B. globosus* snails were collected from uMkhanyakude and Verulam in KwaZulu-Natal Province, South Africa and kept in 2 litre (L) containers filled with filtered pond water. A small polystyrene plate substrate was put in the 2 L containers to provide a surface for snails to lay eggs on. Egg masses deposited on the polystyrene plates and on the walls of the containers were removed as soon as they were noticed and transferred to new containers for hatching at room temperature. The hatchlings were pooled to constitute the first generation (F-1) which was the study population. First generation snails with an average shell height of 3 – 4 mm were used for the experiments.

### Study animals and experimental set up

Four hundred and five laboratory bred F-1 *B. globosus* juveniles were randomly selected and allocated to fifteen 20 L aquaria maintained at five constant temperatures (15.0, 20.0, 25.0, 30.0 and 35.0 °C). Three aquaria were assigned to each experimental temperature and each aquaria contained three 2 L containers holding nine snails. The total number of snails per temperature treatment was 81. Each snail was individually exposed to three miracidia following methods described by Mukaratirwa et al. [22]. The snails were fed *ad libitum* on blanched lettuce and supplemented with Tetramin tropical fish food (Marltons Pet care products). Before the start of the experiments, snails were allowed to acclimatize to their respective experimental temperatures for one week. During the course of the experiments, the containers within the aquaria were rotated weekly to avoid positional effects [23]. The snails were kept in an experimental room with a photoperiod of 12:12 h light–dark cycle.

### Maintenance of constant water temperature

Each aquarium was filled with tap water to provide a water bath for maintaining specific water temperature regulated by a 250 W aquarium heater (JERO 2010 LifeTech) and gas tubes were placed in each aquarium for circulating the water to maintain uniform temperature. The 2 L containers placed in the aquaria were filled with filtered pond water which was changed once a week. A small polystyrene plate was placed in each 2 L container for collecting egg masses. For the pre-determined temperatures of 15.0, 20.0, 25.0, 30.0 and 35.0 °C, the mean (±SE) water temperature that was achieved was: 15.5 ± 0.39, 21.2 ± 0.83, 25.8 ± 0.66, 31.0 ± 0.44 and 36.0 ± 0.35 °C.

### Measurement of fecundity, survival and growth parameters

To evaluate the fecundity of *B. globosus*, we counted the number of egg masses deposited on the polystyrene plates and walls of the containers over a nine-week period. The egg masses were collected, counted and removed from the polystyrene plates and walls of the containers every two days. These were then aggregated to compose a week’s collection.

Snail mortality was recorded daily across the different temperature treatments. Snails were marked as dead if they did not respond to mechanical stimuli while those that showed signs of movements were returned to their experimental temperature treatments [24].

Snail shell heights were measured at the beginning of the experiment and thereafter at two weekly intervals for determination of growth. Fifteen snails from each aquarium (45 snails from each temperature treatment) were randomly selected and their shell heights were measured to the nearest 0.1 mm using vernier callipers (Mitutoyo Corp) following methods described by Doums et al. [25]. The measurement of snail shell height was done over a period of 10 weeks.

### Cercaria counts

From three weeks post infection, all snails were individually exposed to artificial light in shedding tubes to induce cercariae shedding. Snails that shed cercariae were transferred to new 2 L containers and returned to their respective aquarium. The water in the shedding tubes was transferred to corning tubes and 70% ethanol was added to preserve the cercariae for later counting. Cercariae shedding was continued until the 77^th^ day post infection. Counting of cercariae was done in accordance with the methods described by Paull et al. [26] and McClelland [27].

The experiments were terminated at day 80 post infection. This was done to ensure that the longest pre-patent period was catered for and data related to growth and fecundity was collected. Snails that did not shed cercariae from the different experimental temperature treatments were dissected and examined for presence or absence of sporocysts using a dissecting microscope.

### Data analysis

To determine the influence of temperature on host fecundity, an analysis of variance (ANOVA) with Repeated Measures (RM) was used. Egg mass counts were square root transformed to improve homogeneity of variance before being used in the RM ANOVA. Survival analysis was used to determine the influence of temperature on survival time of snails. The survival times were compared between the different temperature groups using the Log Rank (Mantel-cox) tests.

To determine how temperature influenced the onset of cercariae shedding, a proportional hazard test was used. For this analysis, snails exposed to miracidia but which did not shed cercariae were labelled as censored [23].

We estimated the total number of cercariae shed at each experimental temperature as: *T*_*c*_ = ∑_*i* = 1_^*j*^ *P*_*i*_ * *C*_*i*_ * *A*_*i*_ [28] where *T*_*c*_ is the total number of cercariae released, *P*_*i*_ is the proportion of snails shedding cercariae on a particular shedding time point *i, C*_*i*_ is the number of cercariae obtained from the shedding jars of snails shedding cercariae and *A*_*i*_ is the number of snails alive at that temperature treatment at time *i.*

All analyses were conducted using Stata (StataCorp, 2013) and Graphpad Prism 5 for Windows (Graphpad software, San Diego, California, USA).

## Results

### Snail fecundity

**Fig. 1:**
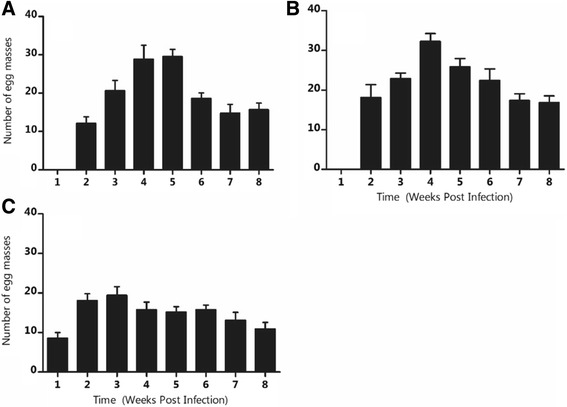
Weekly number of egg masses produced by *Bulinus globosus* maintained at (**a**) 21.2, (**b**) 25.8 and (**c**) 31.0 °C

*Bulinus globosus* snails maintained at average temperatures of 15.5 and 36.0 °C did not lay egg masses while those maintained at 21.2, 25.8 and 31.0 °C did. Time (*P <* 0.001) and temperature (*P <* 0.001) affected the number of egg masses that were deposited. Egg mass production gradually increased during the pre-patent period for all treatment except in the case of 21.2 °C where the decline occured one week before patency (Fig. 1). *Bulinus globosus* snails maintained at 21.2 °C produced 1 254 egg masses while those at 25.8 and 31.0 °C produced 1 359 and 1 015 egg masses, respectively.

### Snail survival

**Fig. 2:**
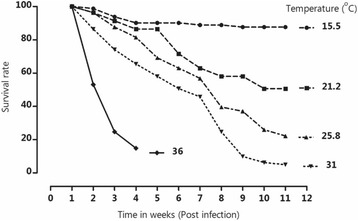
Mortality rates of *Bulinus globosus* maintained at 15.5, 21.2, 25.8, 31.0 and 36.0 °C

As temperature increased, the median survival time reduced (*P* = 0.001). The snails maintained at 36.0 °C had the lowest survival rate of 8.64% while those maintained at 15.5 °C had a survival rate of 87.6% (Fig. 2).

The survival time of snails maintained at 21.2 and 25.8 °C was not significantly different (Log Rank (Mantel-cox) test: *P* = 0.675). However, significant differences (*P <* 0.001) in the survival time were observed between snails maintained at 25.8 and 31.0 °C. Those maintained at 15.5 °C had longer survival time and least hazard ratios as compared to snails maintained at other temperatures (Table 1).

**Table 1:** Comparisons in the survival time of *Bulinus globosus* snails maintained at different temperature levels

Temperature (°C) groups	Chi-square	Hazard ratio	95% *CI* of ratio
15.5 vs 21.2	6.470	0.224*	0.071 – 0.709
15.5 vs 25.8	5.450	0.238*	0.072 – 0.795
15.5 vs 31.0	42.400	0.063***	0.027 – 0.144
21.2 vs 25.8	0.176	0.827	0.339 – 2.014
21.2 vs 31.0	16.050	0.223***	0.107 – 0.465
25.8 vs 31.0	11.920	5.182***	2.459 – 10.92

Significance codes: *0.05, ***0.0001

### Cercariae counts

Increasing temperature had a positive effect on the length of the pre-patent period (*P <* 0.01). Snails maintained at 31.0 °C reached patency after three weeks post infection while those at 21.2 °C reached patency after six weeks. Temperature and time had a significant (Temperature: *P* = 0.0076, Time: *P <* 0.001) influence on the number of cercariae shed from snails. Over time, there was an increase in the number of cercariae shed, followed by a reduction. The estimated mean (± SE) number of cercariae shed at 31.0, 25.8 and 21.2 °C was 829 ± 224.4, 2 409 ± 488.9 and 1 738 ± 409.9 respectively.

### Snail growth

**Fig. 3:**
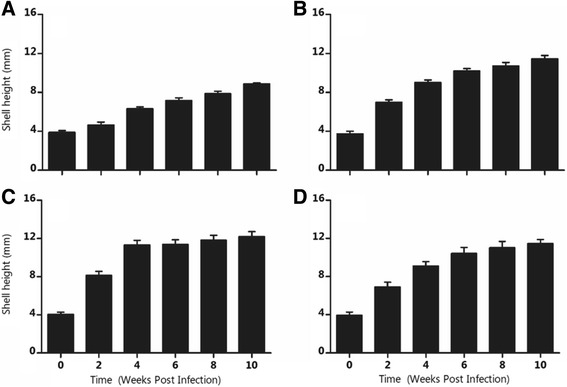
Weekly growth rate of *Bulinus globosus* maintained at (**a**) 15.5, (**b**) 21.2, (**c**) 25.8 and (**d**) 31.0 °C

It was observed that increasing temperature influenced snail growth rate significantly (*P <* 0.001). Snails maintained at 15.5 °C had inhibited growth while those at 31.0 °C had reduced growth (Fig. 3). The results also show that the growth rate of *B. globosus* was higher during the pre-patent period compared to the patent period (Fig. 3).

## Discussion

The reduction in egg mass laying during the patent period shows that trematode infection affects the fecundity of the intermediate host snails. Although egg mass production observed at the three temperature levels seemed to have been accelerated during the early stages of infection, attainment of patency led to a reduction in the egg mass output. This may be due to the rise in the competition for resources by the developing parasites within the host snails. According to Sorensen and Minchella [29], intramolluscan parasite development leads to changes in the host energy allocation patterns. Parasites have also been observed to deplete the hosts’ energy used for gamete production and egg formation which leads to a reduction in the reproduction capacity of the host [30]. Our results indicate that while temperature rise was observed to accelerate gametogenesis [31] leading to increased egg mass output, parasitic infection may result in host fecundity reduction which may have implications on the host population size. This is in agreement with the observations made by Fryer et al. [32], Minchella and Loverde [33], Muñoz-Antoli et al. [34], Jourdane [35] and Paull and Johnson [23]. Temperature may lead to an increase in snail fecundity in some areas with tolerable temperature levels and a reduction in areas with low and very high temperatures. Nevertheless, its net effect may have consequences on the parasite load in the natural environment.

Snail survival was greatly affected by temperature with snails maintained at higher temperature having reduced survival time. All the snails that were maintained at 36.0 °C did not survive beyond the fourth week of the experiment. These findings are similar to those of Woolhouse and Chandiwana [36] who observed that snail mortality increased linearly with temperature above 24.0 °C. The increase in snail mortality may suggest that extreme high temperatures may be unsuitable for the development of snails. With temperature projected to increase due to climate change [37, 38], areas with extreme high temperatures may record reduced snail population sizes [39] which might lead to a reduction in the disease prevalance. Nevertheless, areas with temperatures around 31.0 °C may experience possible disease transmission because of snail availability. Snail longevity at this temperature was reduced. Nonetheless, the snails remained fecund, suggesting that juvenile snails may be introduced to the environment. *Bulinus globosus* snails have been observed to inhabit moist muddy soils or hide under floating aquatic plants [12, 40]. Prolonged hot summer temperatures may lead to drying out of moist areas and aquatic vegetation that provide shelter to snails. This may also lead to a possible reduction in the snail population size [41]. All this suggests that endemic areas with high temperatures may have reduced snail population size which can also impact on the disease transmission.

It was also observed that mortality of snails at 25.8 °C was high but maximal egg mass output was still achieved at this temperature. This increased egg mass output may be attributed to the ability of *B. globosus* to increase its egg mass production as temperature approaches or slightly exceeds 25.0 °C [11, 12]. According to Shiff [13], *B. globosus* maintains its optimal net reproductive rate (*R*_0_) at 25.0 °C. Our results comfirm findings from other studies [11, 13, 42] that suggest that temperatures around and slightly above 25.0 °C may lead to increased egg mass output. Coupled with increased growth rate of snails maintained at 25.8 °C (Fig. 3), this temperature may lead to early development of parasites, thus increasing the disease risks in areas experiencing this temperature. Despite this, the parasites’ effect on the ovotestis of hosts [1, 43] which was observed to lead to castration of snails [23, 44] may offset the positive effect of temperature on snail fecundity.

According to Martens et al. [18] and Knight et al. [45], temperature influences the infection of snails. Furthermore, parasite development within the snails increases as temperature increases while low temperatures inhibit parasite development [23]. An earlier study by Pfluger et al. [46] observed that *B. truncatus* snails exposed to 15.3 °C did not shed cercariae. This is supported by our findings which also showed that at 15.5 °C, *B. globosus* did not develop infection. This means that at this temperature, disease transmission may also be reduced. The optimal temperature range for the transmission of schistosomiasis is 22 – 27 °C [13]. On the other hand, the fact that sufficiently high snail survival rate was observed at 15.5 °C suggests that high snail proliferation may occur when temperature is increased. This may lead to a possible increase in the prevalence of schistosomiasis in certain areas [45]. This may be the case in some endemic areas which experience low temperatures during the winter season. Paull and Johnson [23] observed that cercariae can develop in snails previously maintained at low temperature if the maintenance temperature rises above the minimum cercariae development temperature threshold. This suggests that snails surviving the cold winter seasons in endemic areas may serve as the source of early infection as temperatures become favourable for parasite development.

Our study also suggests that establishment of *S. haematobium* within the host snails may lead to notable alteration in the hosts’ life history traits. Infected snails have been observed to experience significant loss of resources for reproduction [30, 43]. Increased growth of infected snails commonly known as gigantism has been reported [23, 29, 34]. Although gigantism has been associated with trematode infection, variable observations have been made on the effects of parasites on snail growth. According to Chu et al. [47], the growth rate of infected snails was unaffected by trematode infection, while Sorensen and Minchella [48] observed that infection led to a reduction in the growth of snails. Our study observed that infection led to an increase in the growth of snails and this corroborates the findings of Muñoz-Antoli et al. [34] and Sturrock [49]. The increase in the size of infected snails may probably be due to the amount of resources and energy redirected towards somatic growth [50]. On the other hand, the inhibition of snail growth rate at 15.5 °C and its subsequent reduction at 31.0 °C may suggest possible effects of extreme low and high temperatures on snail development and eventual parasite production. Furthermore, Nelson et al. [21] suggest that acclimatization of snails to higher temperature may occur. This may thus change both the population dynamics and infection rates of snails at higher temperatures. The current study observed that snail growth rate was optimum at 25.8 °C and this is similar to the findings of Harrison and Shiff [11]. An increase in snail growth and fecundity around this temperature range may have implications on schistosomiasis prevalence.

## Conclusion

The results suggest that such a mechanistic study model can provide useful information for developing mathematical models that assess and predict the likely changes in the snail populations as a result of temperature change. Although our study has shown some of the direct effects of temperature rise on snail growth rate and parasite production, there is furthermore a need to identify the likely secondary alterations in parasite transmission that may be the outcome of temperature driven changes in the snail-trematode system. The study has also shown that *B. globosus* has a different threshold temperature for reproduction, survival and parasite development and this will have implications on the spread of schistosomiasis. These findings may also be useful in informing schistosomiasis control programmes by identifying new areas that may be targeted for control initiatives on the basis of predicted temperature rises.

## Additional file

Additional file 1: Multilingual abstracts in the five official working languages of the United Nations. (PDF 820 kb)

## Abbreviations

*A*_*i*_: Number of snails alive at that temperature treatment at time *i*
ANOVA: Analysis of Variance
*C*_*i*_: Number of cercariae obtained from the shedding jars of snails shedding cercariae
GLM: Generalised Linear Model
L: Litre
*P*_*i*_: Proportion of snails shedding cercariae on a particular shedding time point *I*
RM: Repeated Measures
*Ro*: Reproductive number
SE: Standard Error
*T*_*c*_: Total number of cercariae
W: Watt

## References

[1] Appleton C, Madsen H (2012). Human schistosomiasis in wetlands in southern Africa. Wetl Ecol Manag.

[2] De Kock K, Wolmarans C (2005). Distribution and habitats of the *Bulinus africanus* species group, snail intermediate hosts of *Schistosoma haematobium* and *S. mattheei* in South Africa. Water SA.

[3] O’keeffe J (1985). Population biology of the freshwater snail *Bulinus globosus* on the Kenya coast. I. Population fluctuations in relation to climate. J Appl Ecol.

[4] De Kock K, Van Eeden J (1986). Effect of programmed circadian temperature fluctuations on population dynamics of Biomphalaria pfeifferi (Krauss). S Afr J Zool.

[5] Appleton C (1978). Review of literature on abiotic factors influencing the distribution and life cycles of bilharziasis intermediate host snails. Malacology Review.

[6] Joubert PH, Pretorius SJ, DeKock KN, Vaneeden JA (1986). Survival of *Bulinus-africanus* (Krauss), *Bulinus-globosus* (Morelet) and *Biomphalaria-pfeifferi* (Krauss) at constant high-temperatures. S Afr J Zool.

[7] Hakalahti T, Karvonen A, Valtonen ET (2006). Climate warming and disease risks in temperate regions–*Argulus coregoni* and *Diplostomum spathaceum* as case studies. J Helminthol.

[8] Pitchford R, Visser P (1969). The use of behaviour patterns of larval schistosomes in assessing the bilharzia potential of non-endemic areas. S Afr Med J.

[9] Shiff C, Husting E (1966). An application of the concept of intrinsic rate of natural increase to studies on the ecology of freshwater snails of the genera *Biomphalaria* and *Bulinus* (Physopsis) in southern Africa. Proc Trans Rhodesian Sci Assoc.

[10] Gordon R, Davey T, Peaston H (1934). The transmission of human bilharziasis in Sierra Leone, with an account of the life cycle of the schistosomes concerned, *S. mansoni* and *S. haematobium*. Ann Trop Med Parasitol.

[11] Harrison A, Shiff C (1966). Factors influencing the distribution of some species of aquatic snails. S Afr J Sci.

[12] Marti H (1986). Field observations on the population dynamics *Bulinus globosus*, the intermediate host of *Schistosoma haematobium* in the Ifakara area, Tanzania. J Parasitol.

[13] Shiff C (1964). Studies on *Bulinus (Physopsis) globosus* in Rhodesia. I. The influence of temperature on the intrinsic rate of natural increase. Ann Trop Med Parasitol.

[14] Stenseth NC, Mysterud A (2002). Climate, changing phenology, and other life history traits: nonlinearity and match–mismatch to the environment. Proc Natl Acad Sci.

[15] Poulin R (2006). Global warming and temperature-mediated increases in cercarial emergence in trematode parasites. Parasitology.

[16] Seppälä O, Jokela J (2011). Immune defence under extreme ambient temperature. Biol Lett.

[17] Pedersen UB, Stendel M, Midzi N, Mduluza T, Soko W, Stensgaard A-S, Vennervald BJ, Mukaratirwa S, Kristensen TK (2014). Modelling climate change impact on the spatial distribution of fresh water snails hosting trematodes in Zimbabwe. Parasit Vectors.

[18] Martens W, Jetten TH, Focks DA (1997). Sensitivity of malaria, schistosomiasis and dengue to global warming. Clim Chang.

[19] Zhou XN, Yang GJ, Sun LP, Hong QB, Yang K, Wang RB, Hua ZH (2002). Potential impact of global warming on the transmission of schistosomiasis. Chinese J Epidemiol.

[20] McCreesh N, Nikulin G, Booth M (2015). Predicting the effects of climate change on *Schistosoma mansoni* transmission in eastern Africa. Parasit Vectors.

[21] Nelson MK, Cruz BC, Buena KL, Nguyen H, Sullivan JT (2016). Effects of abnormal temperature and starvation on the internal defense system of the schistosome-transmitting snail *Biomphalaria glabrata*. J Invertebr Pathol.

[22] Mukaratirwa S, Kristensen TK, Siegismund HR, Chandawana SK (1998). Genetic and morphological variation of populations belonging to the *Bulinus truncatus/tropicus* complex (Gastropoda; Planorbidae) in south western Zimbabwe. J Molluscan Stud.

[23] Paull SH, Johnson PTJ (2011). High temperature enhances host pathology in a snail–trematode system: possible consequences of climate change for the emergence of disease. Freshw Biol.

[24] Joubert P, Pretorius S, De Kock K, Van Eeden J (1984). The effect of constant low temperatures on the survival of *Bulinus africanus*(Krauss), *Bulinus globosus*(Morelet) and *Biomphalaria pfeifferi*(Krauss). S Afr J Zool.

[25] Doums C, Perdieu M-A, Jarne P (1998). Resource allocation and stressful conditions in the aphallic snail *Bulinus truncatus*. Ecology.

[26] Paull SH, Raffel TR, LaFonte BE, Johnson PTJ: How temperature shifts affect parasite production: testing the roles of thermal stress and acclimation. *Functional Ecology*. 2015:1–10. doi:10.1111/1365-2435.12401.

[27] McClelland W (1965). The production of cercariae by Schistosoma mansoni and S. haematobium and methods for estimating the numbers of cercariae in suspension. Bull World Health Organ.

[28] Paull SH, Johnson PT (2014). Experimental warming drives a seasonal shift in the timing of host‐parasite dynamics with consequences for disease risk. Ecol Lett.

[29] Sorensen RE, Minchella DJ (1998). Parasite influences on host life history: *Echinostoma revolutum* parasitism of *Lymnaea elodes* snails. Oecologia.

[30] Gerard C, Theron A (1997). Age/size-and time-specific effects of *Schistosoma mansoni* on energy allocation patterns of its snail host *Biomphalaria glabrata*. Oecologia.

[31] Brackenbury T, Appleton C (1991). Effect of controlled temperatures on gametogenesis in the gastropods *Physa acuta* (Physidae) and *Bulinus tropicus* (Planorbidae). J Molluscan Stud.

[32] Fryer SE, Oswald RC, Probert AJ, Runham NW (1990). The effect of *Schistosoma haematobium* infection on the growth and fecundity of three sympatric species of bulinid snails. J Parasitol.

[33] Minchella DJ, Loverde PT (1981). A cost of increased early reproductive effort in the snail *Biomphalaria glabrata*. Am Nat.

[34] Muñoz-Antoli C, Marín A, Toledo R, Esteban J-G (2007). Effect of *Echinostoma friedi* (Trematoda: Echinostomatidae) experimental infection on longevity, growth and fecundity of juvenile *Radix peregra* (Gastropoda: Lymnaeidae) and *Biomphalaria glabrata* (Gastropoda: Planorbidae) snails. Parasitol Res.

[35] Jourdane J (1982). Effects of hyperinfestations by *Echinostoma togoensis* Jourdane and Kulo, 1981 on growth and survival of *Biomphalaria pfeifferi* snails. Ann Parasitol Hum Comp.

[36] Woolhouse M, Chandiwana S (1990). Population dynamics model for *Bulinus globosus*, intermediate host for *Schistosoma haematobium*, in river habitats. Acta Trop.

[37] Hughes L (2000). Biological consequences of global warming: is the signal already apparent?. Trends Ecol Evol.

[38] Yang G-J, Utzinger J, Sun L-P, Hong Q-B, Vounatsou P, Tanner M, Zhou X-N (2007). Effect of temperature on the development of *Schistosoma japonicum* within *Oncomelania hupensis*, and hibernation of *O. hupensis*. Parasitol Res.

[39] McCreesh N, Booth M (2014). The effect of increasing water temperatures on *Schistosoma mansoni* transmission and *Biomphalaria pfeifferi* population dynamics: an agent-based modelling study. PLoS One.

[40] Woolhouse M, Chandiwana S (1989). Spatial and temporal heterogeneity in the population dynamics of *Bulinus globosus* and *Biomphalaria pfeifferi* and in the epidemiology of their infection with schistosomes. Parasitology.

[41] Bavia ME, Hale LF, Malone JB, Braud DH, Shane SM (1999). Geographic information systems and the environmental risk of schistosomiasis in Bahia, Brazil. AmJTrop Med Hyg.

[42] Shiff CJ, Garnett B (1967). The influence of temperature on the intrinsic rate of natural increase of the freshwater snail *B. pfeifferi*. Arch Hydrobiol.

[43] Probst S, Kube J (1999). Histopathological effects of larval trematode infections in mudsnails and their impact on host growth: what causes gigantism in *Hydrobia ventrosa* (Gastropoda: Prosobranchia)?. J Exp Mar Biol Ecol.

[44] Lafferty KD (1993). Effects of parasitic castration on growth, reproduction and population dynamics of the marine snail *Cerithidea californica*. Mar Ecol - Prog Ser.

[45] Knight M, Elhelu O, Smith M, Haugen B, Miller A, Raghavan N, Wellman C, Cousin C, Dixon F, Mann V, Rinaldi G, Ittiprasert W, Brindley PJ (2015). Susceptibility of snails to infection with schistosomes is influenced by temperature and expression of heat shock proteins. Epidemiol (Sunnyvale, Calif).

[46] Pfluger W, Roushdy M, El-Emam M (1983). Prepatency of *Schistosoma haematobium* in snails at different constant temperatures. J Egypt Soc Parasitol.

[47] Chu K, Massoud J, Sabbaghian H (1966). Host–parasite relationship of *Bulinus truncatus* and *Schistosoma haematobium* in Iran: 3. Effect of water temperature on the ability of miracidia to infect snails. Bull World Health Organ.

[48] Sorensen R, Minchella D (2001). Snail–trematode life history interactions: past trends and future directions. Parasitology.

[49] Sturrock B (1967). The effect of infection with *Schistosoma haematobium* on the growth and reproduction rates of *Bulinus (Physopsis) nasutus productus*. Ann Trop Med Parasitol.

[50] Sousa WP (1983). Host life history and the effect of parasitic castration on growth: a field study of *Cerithidea californica haldeman* (Gastropoda: Prosobranchia) and its trematode parasites. J Exp Mar Biol Ecol.

