# Supplementary material for: Effect of temperature on the Bulinus globosus — Schistosoma haematobium system
Source: Infect Dis Poverty. 2017 May 1;6:57. doi: 10.1186/s40249-017-0260-z (PMC5410706; doi:10.1186/s40249-017-0260-z)
Supplement: Additional file 1: — Multilingual abstracts in the five official working languages of the United Nations. (PDF 820 kb) [file 40249_2017_260_MOESM1_ESM.pdf]

Translation of the abstract into the five official working languages of the United Nations

تأثير درجة الحرارة على نظام بولينس جلوبوسوس-البلهارسيا البولية

كاليندا تشيستر، موسيز ج. تشيمباري، شمشون موكاراتيروا

ملخص

خلفية: بالنظر إلى أن الزيادة في درجة الحرارة قد تغير العلاقات في استضافة الطفيليات، وارتفاع درجات الحرارة المتوقعة نتيجة لارتفاع درجة حرارة الأرض قد تغير أنماط انتقال بعض الأمراض. ومع ذلك، فإن المدى الذي سيحدث عنده ليس مفهوما جيدا.
الأساليب: درسنا نظام مضيف-طفيلي يشمل بولينس جلوبوسوس والبلهارسيا البولية، وقمنا بتقييم تأثير درجة الحرارة على خصوبة الحلزون، والنمو والبقاء ونمو الطفيلي تحت ظروف المختبر.
النتائج: أظهرت نتائجنا أن درجة الحرارة قد يكون لها تأثير غير خطي على خصوبة الحلزون ونمو الحلزون. القواقع التي بقيت في درجة حرارة بين 15.5 و36.0 درجة مئوية لم تنتج كتل البيض في حين أن التي بقيت عند 25.8 درجة مئوية وضعت 344 من كتل البيض أكثر من القواقع التي عاشت في درجة حرارة 31.0 درجة مئوية و105 من كتل البيض أكثر من تلك التي عاشت في 21.2 درجة مئوية. أدى خروج الطفيل إلى انخفاض في إنتاج كتلة البيض بين القواقع. ومع ذلك، حدث انخفاض في خصوبة القواقع التي عاشت في درجة حرارة 21.2 درجة مئوية قبل أن تبدأ القواقع في إطلاق السركاريا. وتسارع تطور الطفيلي في درجات حرارة عالية في القواقع التي عاشت في درجة حرارة 31.0 درجة مئوية لتصل إلى خروج الطفيل بعد ثلاثة أسابيع. وعلاوة على ذلك، كان معدل نمو الحلزون أعلى مستوى عند 25.8 درجة مئوية بينما تحول إلى دون ذلك عند 15.5 درجة مئوية وانخفض عند 31.0 درجة مئوية. ازدادت معدلات وفيات الحلزون بارتفاع درجات الحرارة. القواقع التي بقيت عند 36.0 درجة مئوية عاشت فترة أقصر في حين أن القواقع التي بقيت عند 15.5 درجة مئوية بقيت فترة أطول على قيد الحياة.
الخلاصة: لقد توصلنا إلى أن درجة الحرارة تؤثر على خصوبة ونمو وبقاء الحلزون وعلى نمو الطفيليات في الحلزون وبالتالي تحدد الوقت الذي يستغرقه الطفيلي لإكمال دورة الحياة. هذا له آثار على انتقال البلهارسيا في سياق ظاهرة الاحتباس الحراري.

Translated from English version into Arabic by Mahmoud Sami, through

TRANSLATORS WITHOUT BORDERS

温度对球形小泡螺（*Bulinus globosus*）-埃及血吸虫系统的影响

Kalinda Chester, Moses J. Chimbari, Samson Mukaratirwa

摘要

引言：鉴于温度升高可能会改变宿主-寄生虫的关系，预计由全球变暖带来的预期温度升高可能会改变某些疾病的传播模式。然而，什么程度下发生上述情况还不清楚。
方法：使用球形小泡螺（*Bulinus globosus*）-埃及血吸虫（*Schistosoma haematobium*）的宿主寄生虫系统，在实验室条件下评价了温度对螺的繁殖力、生长、存活与寄生虫发育的影响。
结果：结果表明，温度对螺的繁殖和生长具有非线性的影响。螺在 15.5°C和 36°C不产卵，而在 25.8°C螺的产卵数量比在 31.0 和 21.2°C多 344 和 105 个以上。发育能溢出尾蚴的螺，其产卵量减少。在 21.2°C螺的繁殖力开始下降之前尾蚴溢出，31°C时血吸虫发育加速，3 周后达到能溢出的程度。另外，螺的生长率在 25.8°C最高，在 15.5°C被抑制，在 31°C时降低。气温升高螺死亡率增加。螺在 36°C存活时间最短，而在 15.5°C存活时间最长。

结论：温度影响螺的繁殖力、生长、存活及其体内寄生虫的发育，从而决定了寄生虫完成生命周期的时间。这对全球变暖背景下血吸虫病传播有一定的影响。

Translated from English version into Chinese by Yin-Long Li, edited by Pin Yang

CHINESE CENTER FOR DISEASE CONTROL AND PREVENTION
NATIONAL INSTITUTE OF PARASITIC DISEASES

Effet de la température sur le système *Bulinus globosus – Schistosoma haematobium*

Kalinda Chester, Moses J. Chimbari, Samson Mukaratirwa

Résumé

Contexte : étant donné que l’augmentation de la température peut influer sur le lien hôte–parasite, l’élévation prévue de la température à cause du réchauffement climatique pourrait modifier les schémas de transmission de certaines maladies. Toutefois, on comprend mal dans quelle mesure cela se passera.

Méthodes : en employant un système hôte–parasite impliquant *Bulinus globosus* et *Schistosoma haematobium*, nous avons évalué l’effet de la température sur la fécondité, la croissance, la survie et le développement parasitaire des escargots en conditions de laboratoire.

Résultats : nos résultats montrent que la température peut avoir un effet non linéaire sur la fécondité et la croissance des escargots. Les escargots maintenus à 15,5 et 36,0 °C n’ont pas produit d’amas d’œufs tandis que ceux maintenus à 25,8 °C ont respectivement pondus 344 et 105 amas d’œufs de plus que les escargots à 31,0 °C et 21,2 °C. L’atteinte de la patence a entraîné une réduction de la production d'amas d’œufs parmi les escargots. Cependant, la baisse de la fécondité pour les escargots maintenus à 21,2 °C est intervenue avant que les escargots n’aient commencé à libérer des cercaires. Le développement de parasites était accéléré à des températures élevées, les escargots maintenus à 31,0 °C atteignant la patence au bout de trois semaines. Par ailleurs, le taux de croissance des escargots était supérieur à 25,8 °C tandis qu’il était inhibé à 15,5 °C et réduit à 31,0 °C. L’augmentation de la température a engendré celle du taux de mortalité des escargots. Les escargots maintenus à 36,0 °C présentaient la durée de survie la plus courte alors que ceux maintenus à 15,5 °C étaient associés à la durée de survie la plus longue.

Conclusion : nous avons conclu que la température joue sur la fécondité, la croissance, la survie et le développement parasitaire de l’escargot, et détermine donc la durée nécessaire au parasite pour parvenir au terme du cycle de vie. Cela a des implications sur la transmission de la schistosomiase dans le contexte du réchauffement climatique.

Translated from English version into French by Agathe Lefèvre, through

TRANSLATORS
WITHOUT BORDERS

Температурное воздействие на огранизмы *Bulinus globosus – Schistosoma haematobium*

Калинда Честер, Мозес Дж. Чимбари, Сэмсон Мукаратирва

Реферат

Предыстория: Учитывая, что повышение температуры может изменять взаимосвязи между хозяином и паразитом, ожидаемое повышение температуры вследствие глобального потепления может изменить модели передачи некоторых болезней. Однако, масштабы этого процесса не вполне понятны.

Методы: Используя систему хозяин-паразит, включающую *Bulinus globosus* и *Schistosoma haematobium,* мы оценивали температурное воздействие на фертильность, рост, выживание улиток и развитие в них паразитов в лабораторных условиях.

Результаты: Наши результаты показывают, что температура может оказывать нелинейное воздействие на фертильность и рост улиток. Улитки, содержавшиеся при температурах 15,5 и 36,0 °C, не производили кладку яиц; а улитки, содержавшиеся при температуре 25,8 °C, откладывали на 344 и 105 яиц больше, чем улитки при температурах 31,0 °C и 21,2 °C соответственно. Достижение раскрытого состояния вело к снижению кладки яиц среди улиток. Однако, снижение фертильности у улиток, содержавшихся при 21,2 °C, происходило до начала откладывания церкариев. Развитие паразитов ускорялось при высоких температурах у улиток, содержавшихся при 31,0 °C и достигших раскрытого состояния через три недели. Более того, скорость роста улиток была наивысшей при 25,8 °C, замедлялась при 15,5 °C и сокращалась при 31,0 °C. При повышении температуры увеличивалась смертность улиток. Улитки, содержавшиеся при 36,0 °C, имели самое короткое время выживания, тогда как улитки, содержавшиеся при 15.5 °C, имели самое длительное время выживания.

Выводы: По нашим выводам, температура влияет на фертильность, рост, выживаемость и развитие паразитов в улитках и, следовательно, обусловливает время, необходимое для завершения жизненного цикла паразитов. В контексте глобального потепления этот факт имеет последствия для передачи шистосомоза.

Translated from English version into Russian by Tatyana Johnson, through

TRANSLATORS
WITHOUT BORDERS

Efecto de la temperatura en el sistema *Bulinus globosus – Schistosoma haematobium*

Kalinda Chester, Moses J. Chimbari, Samson Mukaratirwa

Reseña

Antecedentes: Dado que el incremento en la temperatura puede alterar las relaciones huésped-parásito, el aumento anticipado de la temperatura debido al calentamiento global podría cambiar los patrones de transmisión de ciertas enfermedades. Sin embargo, aún no se comprende bien hasta qué

punto esto pueda ocurrir.
Métodos: Utilizamos un sistema huésped-parásito que involucró al *Bulinus globosus* y al *Schistosoma haematobium* y evaluamos el efecto de la temperatura en la fecundidad del caracol, su crecimiento y supervivencia así como el desarrollo del parásito en condiciones de laboratorio.
Resultados: Nuestros resultados muestran que la temperatura quizá tenga un efecto no lineal en la fecundidad del caracol y su crecimiento. Los caracoles mantenidos a 15.5 y a 36 ºC no produjeron masas de huevos, mientras que los mantenidos a 25.8 ºC pusieron 344 y 105 más de masas de huevos que los caracoles a 31 ºC y a 21.2 ºC, respectivamente. Lograr la patencia llevó a que se redujera la producción de masa de huevos entre los caracoles. Sin embargo, la disminución de la fecundidad en caracoles mantenidos a 21.2 ºC sucedió antes de que los caracoles comenzaran a liberar cercarias. El desarrollo de parásitos se aceleró a temperaturas altas en caracoles mantenidos a 31 ºC y se alcanzó la patencia después de tres semanas. Más aún, la tasa de crecimiento de los caracoles fue la más alta a los 25.8 °C, mientras que se inhibió a los 15.5 ºC y se redujo a los 31 ºC. El aumento de la temperatura, incrementó la tasa de mortalidad de los caracoles. Los caracoles mantenidos a 36 ºC tuvieron el tiempo más corto de supervivencia, mientras que aquellos mantenidos a 15.5 ºC alcanzaron el período más largo de supervivencia.
Conclusión: Concluimos que la temperatura influye en la fecundidad, crecimiento, supervivencia y desarrollo del parásito en el caracol, por lo tanto, determina el tiempo que le lleva al parásito completar el ciclo de vida. Esto tiene consecuencias en la transmisión de la esquistosomiasis en el contexto del calentamiento global.

Translated from English version into Spanish by LidiaN, through

TRANSLATORS
WITHOUT BORDERS
